# Feasibility Study of Nivolumab in Combination with Carboplatin Plus Paclitaxel and Concurrent Thoracic Radiation in Patients with Untreated Unresectable Locally Advanced Non-Small Cell Lung Cancer

**DOI:** 10.3390/cancers16183127

**Published:** 2024-09-11

**Authors:** Yuta Yamanaka, Takayo Ota, Yutaka Masuoka, Yuki Takeyasu, Satoaki Nakamura, Masaaki Terashima, Hiroshige Yoshioka, Masahiro Fukuoka, Takayasu Kurata

**Affiliations:** 1 Department of Thoracic Oncology, Kansai Medical University Hospital, 2-5-1 Shinmachi, Hirakatashi 573-1010, Osaka, Japan; 2 Medical Oncology, Izumi City General Medical Center, 4-5-1 Wakechou, Izumishi 594-0073, Osaka, Japan; takayo.ota@gmail.com (T.O.);; 3 Department of Radiology, Izumi City General Medical Center, 4-5-1 Wakechou, Izumishi 594-0073, Osaka, Japan; kmjyuta@yahoo.co.jp; 4 Department of Radiology, Kansai Medical University Hospital, 2-5-1 Shinmachi, Hirakatashi 573-1010, Osaka, Japan

**Keywords:** feasibility study, nivolumab, chemoradiotherapy, unresectable locally advanced non-small cell lung cancer

## Abstract

**Simple Summary:**

Lung cancer remains a leading cause of death despite advancements in treatment. A recent phase III trial has shown that adding an immune checkpoint inhibitor (ICI) to standard chemoradiation therapy (CCRT) improves survival in patients with advanced lung cancer. A sub-analysis of the same study also showed that shortening the interval between CRT and ICI increased the clinical benefits. Therefore, a feasibility study of nivolumab plus CCRT was conducted in Japanese patients with unresectable locally advanced NSCLC. We enrolled 12 patients and examined 10 for dose-limiting toxicities (DLT). The treatment was considered feasible if DLT occurred in three or fewer of the ten evaluable patients, meeting the study criteria. The findings suggest that nivolumab combined with CCRT is a safe and promising treatment option for patients with previously untreated unresectable locally advanced NSCLC, potentially improving their chances of survival.

**Abstract:**

Despite advancements in diagnosing and treating non-small cell lung cancer (NSCLC), the prognosis remains poor. Immune checkpoint inhibitors have shown promise in enhancing survival rates. Therefore, this study aimed to investigate the safety of nivolumab administration with concurrent chemoradiation therapy (CCRT) in patients with unresectable locally advanced NSCLC. Twelve patients with unresectable locally advanced NSCLC at Kansai Medical University Hospital and Izumi City General Medical Center were enrolled from May 2018 to September 2020. They received nivolumab (360 mg) tri-weekly twice, weekly carboplatin (AUC 2 min × mg/mL) and paclitaxel (40 mg/m^2^) for 6 weeks, and thoracic radiotherapy (60 Gy/30 fractions), followed by maintenance nivolumab therapy (360 mg, tri-weekly) for 6 months. The primary endpoint was incidence of dose-limiting toxicities (DLTs), and the secondary endpoints included safety, response rate, progression-free survival (PFS), overall survival (OS), 2-year survival rate, and treatment completion rate. Three patients completed the protocol. Nine discontinued due directly to interstitial pneumonia (three) and pneumonia (one). Ten patients (83.3%) experienced a grade 3 or higher event, of which three (25%) experienced a grade 4 or higher event, and of these, one (8.3%) experienced a grade 5 event. Three patients experienced DLTs. Concurrent nivolumab with CCRT was tolerated in unresectable locally advanced NSCLC, which offers potential treatment benefits.

## 1. Introduction

Despite advancements in the early diagnosis and treatment of non-small cell lung cancer (NSCLC), its prognosis remains poor. Most patients present with distant metastases at the time of diagnosis, whereas approximately one-third of the patients are diagnosed with stage III cancer, classified as locally advanced NSCLC. For such cases with good performance status (PS), concurrent chemoradiotherapy (CCRT) has long been the traditional standard curative treatment. However, despite the introduction of novel chemotherapeutic agents (e.g., pemetrexed) and improvements in radiotherapy technology, the median survival time (MST) and 5-year survival rates remain unchanged over the last 20 years [1,2,3,4,5]. This underscores the urgent need for novel therapeutic approaches.

In recent years, immune checkpoint inhibitors (ICIs), particularly anti-programmed death 1 (PD-1) (nivolumab, pembrolizumab) and anti-programmed death ligand 1 (PD-L1) (durvalumab, atezolizumab) blockers, have been introduced for the treatment of advanced NSCLC, resulting in the long-term survival in some cases. Preclinical evidence suggests a synergistic effect between cytotoxic chemotherapy and ICIs through immunogenic cell death (ICD) or a drug-induced increase in antigenicity [6]. As such, several phase III studies comparing a combination therapy of chemotherapy and ICIs versus chemotherapy alone have been conducted in treatment-naïve patients with advanced NSCLC, demonstrating significantly prolonged overall survival (OS) in those who received combination therapy [7,8,9]. Similarly, radiation therapy appears to have a synergistic effect with ICIs through ICD and transient upregulation of PD-L1 [10,11,12]. A previous randomized phase III study showed that, compared with the placebo, durvalumab significantly improved progression-free survival (PFS) (hazard ratio [HR] = 0.52, 95% confidence intervals [CIs]: 0.42–0.65, *p* < 0.0001) and OS (HR = 0.68, 95% CIs: 0.53–0.87, *p* = 0.0025) in patients with unresectable stage III NSCLC who had not progressed after CRT [13,14]. Furthermore, since no safety concerns have emerged in these studies, durvalumab has become the standard regimen for consolidation therapy in this cohort. Interestingly, a sub-analysis of the previous study highlighted the enhanced clinical benefits with a shorter interval between CRT and durvalumab. However, the optimal regimen and timing for combined ICI therapy and CRT remain undefined.

Theoretically, subsequent/closer or simultaneous administration seems to be suitable for maximizing the synergistic effect of ICI and CRT. Therefore, the present study aimed to evaluate the efficacy of nivolumab administration with CCRT. However, given the lack of safety data regarding this combination therapy, we first conducted a feasibility study of this regimen among Japanese patients with unresectable locally advanced NSCLC.

## 2. Materials and Methods

### 2.1. Patients

The inclusion criteria for this study were as follows: histologically and/or cytologically confirmed stage III unresectable NSCLC, treatment-naïve status, age 20–74 years, Eastern Cooperative Oncology Group PS of 0 or 1, and adequate organ function (neutrophil count ≧ 1500/mm^3^, hemoglobin level ≧ 9.0 g/dL, platelet count ≧ 100,000/mm^3^, serum transaminase level ≦ 100 IU/L, total bilirubin level ≦ 1.5 mg/dL, serum creatinine level ≦ 1.2 mg/dL, partial pressure of oxygen [PaO_2_] ≧ 70 Torr, or oxygen saturation [SpO_2_] ≧ 93%). All included patients were hospitalized during the concurrent phase for safety confirmation.

The exclusion criteria for the study were as follows: active concomitant malignancy, serious infection, severe comorbidities (e.g., uncontrollable diabetes, uncontrollable angina pectoris, diverticulitis, symptomatic gastrointestinal ulcer, emphysema, chronic bronchitis, bronchial asthma, drug-induced interstitial pneumonia, active autoimmune disease, and grade 2 or higher peripheral neuropathy), and evidence of interstitial pneumonia or pulmonary fibrosis on chest computed tomography (CT).

After 9 patients (11 total cases) were enrolled, the study encountered a treatment-related death due to interstitial pneumonia. Therefore, the protocol was amended before enrolling the last patient for a more accurate assessment of treatment safety, as follows: (1) a 3-week recovery period was given prior to starting treatment in patients with non-serious infectious disease; (2) the V20 lung volume limit was revised from ≦ 35% to ≦ 30%. As such, the final criteria for candidates for curable irradiation were as follows: absence of intra-lobe metastasis, unilateral atelectasis, and contralateral hilar lymph node metastasis; V20 ≦ 30%; and approval from radiation oncologists.

### 2.2. Study Design and Treatment

This was a feasibility study that evaluated the safety of nivolumab administration with CCRT at two institutions (Kansai Medical University Hospital and Izumi City General Hospital) in Japan, with only one dose level in ten patients. Specifically, patients received tri-weekly doses of nivolumab (360 mg/body) twice, plus weekly doses of carboplatin (CBDCA) (AUC 2 min × mg/mL) [15] and paclitaxel (PTX) (40 mg/m^2^) for 6 weeks, which was concurrently given with definitive thoracic radiotherapy (60 Gy/30 fractions). A maintenance nivolumab regimen (360 mg/body, day 1, tri-weekly) was given for 6 months thereafter. At the discretion of the physician, two cycles of tri-weekly CBDCA (AUC 5 min × mg/mL) and PTX (200 mg/m^2^) may be given as consolidation therapy. For thoracic radiation therapy (TRT), a three-dimensional treatment plan (target reference point prescription) or intensity-modulated radiotherapy (IMRT: D95 prescription) can be selected based on institutional practices. Concurrent TRT started from day 1, with daily 2-Gy exposures for 5 days a week for a total dose of 60 Gy in 30 fractions. The allowable treatment period was 63 days, accounting for rest and postponement.

The primary endpoint was incidence of dose-limiting toxicities (DLTs) within 90 days of treatment initiation, as indicated in the Common Terminology Criteria for Adverse Events version 4.0 [16]. The secondary endpoints included safety (rate of adverse events [AEs]), response rate, PFS, OS, 2-year survival rate, and treatment completion rate (concurrent phase). The criteria for DLTs included the following factors: grade 4 non-hematologic toxicity, grade 3 non-hematologic toxicity persisting for 3 consecutive days despite adequate supportive treatment, grade 4 neutropenia lasting ≧ 8 days, febrile neutropenia, grade 4 thrombocytopenia or grade 3 thrombocytopenia requiring platelet transfusion, grade 3 or higher interstitial lung disease (e.g., radiation pneumonitis), any AEs hindering the completion of thoracic radiotherapy within 63 days of radiotherapy initiation, any AEs preventing nivolumab administration two times or more—or preventing three or more rounds of CCRT—and AEs that physicians deemed too difficult for continued treatment. The treatment is deemed feasible if DLTs occurred in no more than three of the ten evaluable cases, indicating that the evaluation of efficacy can now be considered.

In the concurrent phase, patients could resume chemotherapy on day 8 or later if they had good PS; neutrophil count ≧ 1000/mm^3^; platelet count ≧ 75,000/mm^3^; serum creatinine level ≦ 1.2 mg/dL; and absence of fever, radiation therapy discontinuation, and non-hematological toxicity of grade 2 or less. If these criteria were not met, both drugs were skipped. Similarly, nivolumab can be administered unless there is a presence of grade 1 or higher interstitial pneumonia; grade 2 or higher colitis, diarrhea, neurotoxicity, and creatinine increase; or if other grade 3 or higher toxicities were present. Moreover, PTX dose reductions (30 mg/m^2^ or 20 mg/m^2^) were implemented if patients presented with the following characteristics: neutrophil count ≦ 500/mm^3^ persisting for >8 days, platelet count ≦ 25,000/mm^3^, platelet count ≦ 50,000/mm^3^ requiring platelet transfusion, neutropenic fever, PTX discontinuation of at least two times, and grade 3 or higher non-hematological toxicity. Meanwhile, no dose adjustments were implemented for CBDCA and nivolumab. For radiotherapy, it was necessary to confirm an SpO_2_ ≧ 90% on room air before each irradiation. In cases where SpO_2_ did not reach 90%, the following criteria were checked: grade 4 neutropenia and thrombocytopenia, body temperature ≧ 38 °C associated with infection, grade 3 esophagitis and dermatitis, and an interstitial pneumonia or PaO_2_ decrease of ≧ 20 mmHg. If any of these criteria were positive, radiotherapy was postponed until there was an improvement of the condition.

In the consolidation phase, chemotherapy could be administered if patients had good PS, neutrophil count ≧ 1500/mm^3^, white blood cell count ≧ 3000/mm^3^, platelet count ≧ 100,000/mm^3^, serum creatinine level ≦ 1.2 mg/dL, and absence of infectious fever or non-hematological toxicity of grade 2 or less. If these criteria were not met, both drugs were delayed for 1 week, with consideration for protocol discontinuation for 3-week postponements. However, in patients who could not receive chemotherapy due to AEs, but who had recovered to meet the nivolumab administration criteria, only nivolumab maintenance therapy was given. Similarly, consolidation nivolumab can be administered unless there is a presence of grade 1 or higher interstitial pneumonia; grade 2 or higher colitis, diarrhea, neurotoxicity, and creatinine increase; or if other grade 3 or higher toxicities were present. In cases where there was interstitial pneumonia recovery, nivolumab was restarted, but subsequent administration was discontinued if the condition recurred.

Although prohibited until disease progression, post hoc treatment was permitted if it was necessary, considering the patient’s wishes and benefits.

### 2.3. Evaluations

For pretreatment evaluation, a comprehensive history assessment and physical examination were conducted for each patient to collect data regarding their background information, PS, histologic diagnosis, tumor staging, evaluable and non-evaluable lesions, presence of complications, and smoking status. Testing for gene alterations, such as epidermal growth factor receptor (EGFR) mutation, was not mandatory. In addition to routine blood tests and imaging, specific clinical tests were performed to assess thyroid function, rheumatoid factor, antinuclear antibody, surfactant protein-D, Krebs von den Lungen-6 (KL-6) levels, pulmonary function, electrocardiography, and PaO_2_ or arterial blood gas. After treatment initiation, physical examination, blood tests, and chest radiography were performed on the day of chemotherapy and/or nivolumab initiation to evaluate safety. Imaging tests were then performed every 6 weeks for monitoring.

PFS was defined as the time from enrollment to the date of disease progression or death, whereas OS was defined as the time from enrollment to death.

### 2.4. Statistical Analysis

PFS and OS were analyzed using Kaplan–Meier analysis. Median values and corresponding 95% confidence intervals (CIs) were generated using the Brookmeyer–Crowley method. All analyses were performed using JMP Pro V.17 (SAS Institute Inc., Cary, NC, USA), and the overall response rate and disease control rate were evaluated using Response Evaluation Criteria in Solid Tumors, version 1.1.

## 3. Results

**Figure 1:**
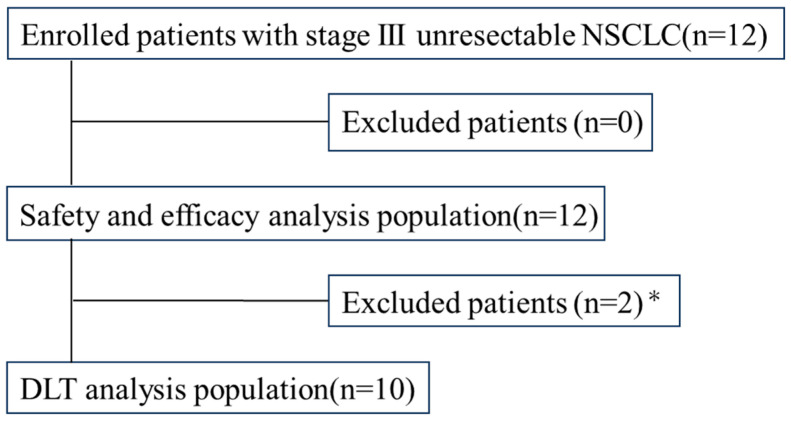
CONSORT diagram of this study. * One patient required >10 mg/day of systemic corticosteroids for a non-protocol-related pulmonary infection, while the other patient withdrew from the study.

A total of 12 patients from Kansai Medical University Hospital and Izumi City General Medical Center were enrolled in the study from May 2018 to September 2020. The CONSORT diagram is shown in Figure 1. While all patients were evaluated for safety and efficacy, two patients were excluded from DLT analysis due to the required discontinuation of the protocol treatment. One patient required systemic corticosteroid doses > 10 mg/day for a pulmonary infection unrelated to the protocol treatment, and the other patient withdrew from the study. Therefore, only 10 patients underwent DLT analysis.

### 3.1. Patient Characteristics

Patient characteristics are summarized in Table 1. Among them, 11 patients were men (92%), and the median age was 65.5 years (range: 53–74 years). Regarding tumor histology, five patients had non-squamous cell carcinoma (42%), and seven had squamous cell carcinoma (58%). Regarding EGFR mutation status, one patient showed positive results (Exon19del), and one showed negative, while the status was unknown in the remaining ten patients. Regarding smoking history, three patients (25%) were current smokers, and nine (75%) had a history of smoking.

### 3.2. Safety

Among the twelve patients, only three completed the protocol. Regarding the reason for discontinuation of the protocol in the nine patients, four showed disease progression, four showed an inability to tolerate the protocol treatment (three with interstitial pneumonia and one with pneumonia), and one showed patient withdrawal. All 12 patients experienced treatment-related AEs, as listed in Table 2. Notably, fifteen grade 3 events were noted in ten patients (83.3%), three grade 4 events were noted in three patients (25.0%), and one grade 5 event was noted in one patient (8.3%). Based on specific AEs, leukopenia of grades 3–5 was noted in three (25%), one (8.3%), and zero patients; neutropenia of grades 3–5 was noted in two (16.7%), two (16.7%), and zero patients; and interstitial pneumonia of grades 3–5 was noted in two (16.7%), zero, and one (8.3%) patient, respectively. Other grade 3 AEs included pneumonia in three patients (25%), as well as diarrhea, femoral neck fracture, and decreased appetite in one patient (8.3%) each. The most common treatment-related AEs below grade 2 were non-hematological toxicities, nausea, constipation, rash, fatigue, and radiation esophagitis. As for the DLT, only three of the ten patients fulfilled the criteria, with two occurring during the evaluation period and one beyond the evaluation period.

### 3.3. DLT Cases

#### 3.3.1. Case 1

A 74-year-old man was diagnosed with cT4N2M0 Stage IIIB squamous cell carcinoma located at the upper lobe of the right lung. He had a PS of 0 and a V20 of 26.16%. He was diagnosed with grade 3 interstitial lung disease (ILD) on day 83, from the start of treatment, and was admitted to the hospital. CT showed an organizing pneumonia pattern, and treatment with 0.5 mg/kg of prednisolone (30 mg/body) was started. He was discharged on day 95, and the prednisolone regimen was gradually tapered when his dose reached 30 mg.

#### 3.3.2. Case 2

A 67-year-old man was diagnosed with cT4N3M0 Stage IIIC squamous cell carcinoma located at the upper lobe of the left lung. He had a PS of 0 and a V20 of 25.97%. He was diagnosed with grade 3 ILD on day 95, from the start of treatment, and was admitted to the hospital. His CT revealed an organizing pneumonia pattern, and treatment with 1.0 mg/kg of prednisolone (60 mg/body) was started. He was discharged on day 122, from the start of treatment, and prednisolone was eventually discontinued due to improved ILD. He was then allowed to resume chemotherapy without relapse of ILD.

#### 3.3.3. Case 3

A 72-year-old man was diagnosed with cT2bN2M0 Stage IIIA squamous cell carcinoma located at the upper lobe of the lung. He had a PS of 0 and a V20 of 23.55%. He was diagnosed with grade 3 ILD on day 80 from the start of treatment, and was admitted to the hospital. His CT showed an organizing pneumonia pattern, and treatment with 0.5 mg/kg of prednisolone (30 mg/body) was initiated. He was discharged on day 97 from the start of treatment, and prednisolone was discontinued. However, the appearance of bone and liver metastases on day 198 from the start of treatment confirmed lung cancer recurrence. He underwent subsequent chemotherapy (CBDCA plus nano albumin-bound Paclitaxel [nab-PTX] or a combination of tegafur, gimeracil, and oteracil potassium [TS-1]), but ILD recurrence was observed on day 365, from the start of treatment, prompting initiation of methylprednisolone (1000 mg). Unfortunately, his condition did not improve, resulting in his death on day 382 from the start of treatment. The cause of death was determined to be grade 5 ILD relapse.

In summary, our DLT cases comprised two patients with grade 3 interstitial pneumonia, who were responsive to steroid therapy, and one patient with grade 5 interstitial pneumonia, who died due to complications of his condition.

### 3.4. Treatment Efficacy

**Figure 2:**
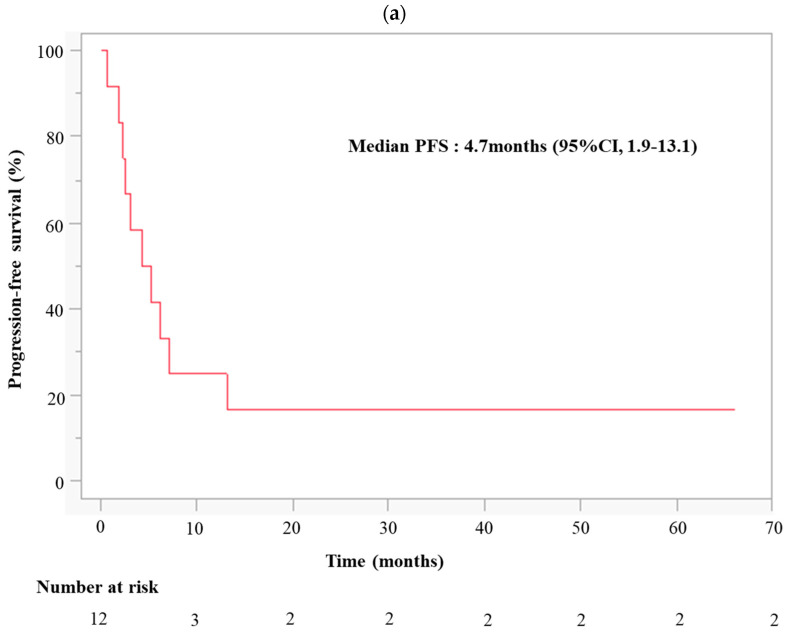

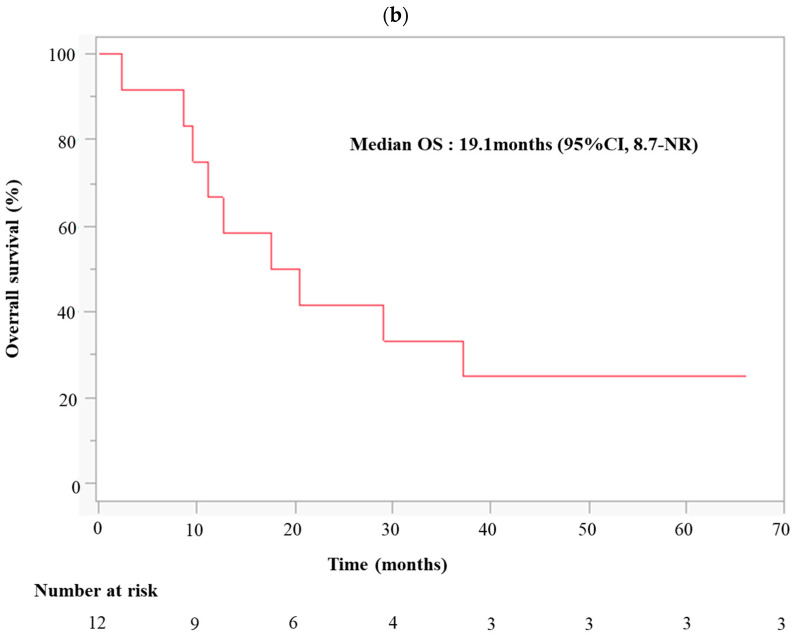
Kaplan–Meier estimates of (**a**) PFS and (**b**) OS in 12 patients who received nivolumab and CCRT. Abbreviations: PFS, progression-free survival; OS, overall survival; CCRT, concurrent chemoradiation therapy.

Among the evaluable patients, seven achieved partial response (PR), and two maintained stable disease (SD), resulting in an overall response rate and disease control rate of 58.33% (95% CIs: 27.67–84.83) and 75.00% (95% CIs: 42.81–94.51), respectively (Table 3). Due to early discontinuation in the three cases, the best overall response could not be determined. The median follow-up was 9.3 months (range: 1.5–50.9 months). The median PFS was 4.7 months (95% CIs: 1.9–13.1 months), while the median OS was 19.1 months (95% CIs: 8.7–not reached) (Figure 2). Furthermore, the 2-year survival rate was 16.67% (95% CIs: 2.09–48.41), with two of twelve patients surviving after 2 years, and the treatment completion rate was 83.3%.

## 4. Discussion

In this feasibility study, we examined the efficacy of nivolumab administration with CCRT. CCRT (e.g., platinum-based agents) and radiation therapy with subsequent ICI administration remain the standard of care for unresectable locally advanced NSCLC. Preclinical models have reported immune mechanisms of tumor control by radiation, wherein local radiotherapy can promote anti-tumor immune responses [17,18]. Moreover, the KEYNOTE-001 and PACIFIC trials demonstrated significant improvement in PFS and OS with ICI use in patients initially treated with radiation therapy [19,20]. Other studies have similarly reported the synergistic effects of combined ICI and radiotherapy regimens [21], and their abscopal effect has been demonstrated in clinical practice [22]. Furthermore, studies with mice models indicate increased efficacy with ICIs and CCRT compared to sequential administration [23]. Given these findings, there are expectations for the application of CCRT and ICI regimens in cancer treatment. However, prior to testing the efficacy of this combination, it is necessary to first investigate the safety of CCRT and ICI, particularly with immune-related AEs.

In this study, we investigated the feasibility of nivolumab administration in combination with CBDCA plus PTX and CCRT in treatment-naïve patients with unresectable locally advanced NSCLC. DLT analysis was performed in ten of the twelve patients, noting the condition in three cases that can be classified as DLTs. Specifically, we noted two cases of grade 3 and one case of grade 5 interstitial pneumonia, indicating that the protocol was tolerable (DLT rate: 30%). In Case 3, referring to the patient with grade 5 ILD, rapid improvement with prednisolone, lack of recurrence after discontinuation, and administration of chemotherapy only after relapse all suggest potential post-treatment factors in ILD development. Therefore, it is reasonable to conclude that this study was tolerated. According to a meta-analysis of 800 cases, high V20 levels were associated with an increased risk of pneumonitis [24]. Moreover, rapid pneumonitis development after radiation was highly associated with severe disease [25]. Another study also showed that a V20 > 26% indicated a 50% risk of pneumonia, whereas a V20 <26% indicated a 27% risk [26]. In this study, the V20 values of the DLT cases were 23%, 25%, and 26%, with Case 3 exhibiting the smallest V20 value. Since V20 was not the only risk of ILD, and because Case 3 was the ninth enrolled patient, we revised the clinical protocol for a stricter selection of eligible patients, as explained in the Section 2. These changes also support the possibility of post-treatment factors in the development of severe ILD. Nevertheless, since all the DLTs in this study manifested as interstitial pneumonia, we believe that stricter practices are needed for the evaluation of lung function and the extent of radiation exposure before starting the protocol treatment.

Several previous studies exist. The NICOLAS trial, conducted by Peters et al. in 2019 [27,28], employed a similar phase II design that evaluated the safety and efficacy of CRT plus nivolumab for unresectable locally advanced NSCLC. In their trial, the chemotherapy regimen utilized cisplatin or carboplatin combined with vinorelbine, etoposide, or pemetrexed. Among the seventy-seven patients in their study, they noted nine cases (11.7%) of grade 3 or higher pneumonitis, and one case of grade 5 pneumonitis, wherein six of these cases occurred within 6 months after radiotherapy. Regarding their toxicity profile, they reported a total of sixty-one serious AEs by grade, including fifteen grade 2 cases (24.6%), twenty-nine grade 3 cases (47.5%), eight grade 4 cases (13.1%), and eight grade 5 cases (13.1%). Comparing our findings with theirs, the toxicity profile was found to be similar, although our study reported a higher rate of interstitial pneumonia. In addition, our study reported one grade 5 case (8.3%) compared to the eight grade 5 cases (13.1%) in the NICOLAS study, suggesting that treatment-related death may not be significantly elevated in our approach. The KEYNOTE-799 trial, conducted by Salma K. Jabbour, et al. in 2020 [29,30], employed a similar phase I and II design that evaluated the safety and efficacy of CRT plus pembrolizumab for unresectable locally advanced NSCLC. Twenty-three patients were enrolled in the study, and 21 patients received at least one dose of pembrolizumab. Two patients died before receiving pembrolizumab. The incidence of Grade 2 or higher immune-related adverse events was 14 cases (66.7%), and Grade 2 or higher pneumonia occurred in 7 cases (33%). One of these cases was Grade 5. Although this study did not show DLTs and is therefore considered to be tolerated. However, although the number of cases differs from our study and the NICOLAS study, this study also showed a certain number of cases of pneumonia, suggesting that great caution should be exercised in the occurrence of pneumonia in any study.

Analyzing the present study’s efficacy, our median PFS was 7.1 months, which falls below that in the RTOG0617 (60 Gy group: 12.0 months), PROCLAIM (cisplatin plus pemetrexed group: 11.4 months), PACIFIC (durvalumab group: 16.9 months), and NICOLAS trials (12.7 months) [31,32]. Similarly, our median OS of 17.7 months was lower than their respective OS rates of 28.7, 26.8, 47.5, and 38.8 months. Although our trial proves the safety of nivolumab with CCRT, our reported efficacy was similar or slightly inferior to other studies. Several limitations could have contributed to this discrepancy, such as the limited patient pool, early discontinuations due to AEs and treatment-related deaths, and the inclusion of a larger proportion of Stage IIIB and IIIC patients compared to other trials. Radiation and immune checkpoint blockade are known to have synergistic effects, and while adding nivolumab to CCRT may add to the therapeutic effect, this study was conducted first to determine its safety, as the overlap of radiation, immune checkpoint inhibitors, and chemotherapy may lead to an increase in serious adverse events. However, since this study showed that this treatment is tolerated in Japanese patients, it is possible to use the same concept treatment in subsequent phase II or phase III studies.

## 5. Conclusions

This study determined the nivolumab administration with CCRT as a tolerated regimen among treatment-naïve Japanese patients with unresectable locally advanced NSCLC. However, the three cases of AE-related early discontinuation warrant further caution regarding the feasibility and safety of future phase II/III trials. In terms of efficacy, this regimen shows potential as a first-line treatment with high anti-tumor effects. However, further evaluation with large-scale phase II/III trials is needed to validate these findings. Nonetheless, this study demonstrated the safety of ICI therapy with CCRT in Japanese patients, which should serve as the basis for similar studies in the future.

## Figures and Tables

**Table 1:** Patient characteristics.

No. of patients		12
Age: year	Median age	65.5
	range	53–74
Sex	Male/Female	11/1
Performance Status	0/1	11/1
Histology	Adeno/Squamous	5/7
Stage	IIIA/IIIB/IIIC	5/6/1
EGFR gene mutation	(+)/(−)/unknown	1/1/10
Smoking history	current/ former/never	3/9/0

**Table 2:** Major treatment-related adverse events within all durations (n = 12).

	Grade					
	1	2	3	4	5	All Grades
Leukopenia	1	4	3	1	0	9
Neutropenia	0	0	2	2	0	4
Lymphopenia	0	1	1	0	0	2
Anemia	3	4	0	0	0	7
Thrombocytopenia	0	1	1	0	0	2
Radiation esophagitis	3	3	0	0	0	6
Radiation pneumonitis	3	3	0	0	0	6
Pneumonia	0	1	2	0	0	3
Interstitial pneumonia	0	0	2	0	1	3
Nausea	7	1	0	0	0	8
Vomiting	2	0	0	0	0	2
Constipation	7	1	0	0	0	8
Diarrhea	2	1	1	1	0	4
Fatigue	6	2	0	0	0	8
Rash	5	1	0	0	0	6
Decreased appetite	3	2	1	0	0	6
Dysgeusia	4	0	0	0	0	4
Insomnia	4	0	0	0	0	4
Depilation	2	1	0	0	0	3
Dizziness	3	0	0	0	0	3

**Table 3:** Treatment response.

Objective Response	
CR	0 (0)
PR	7 (58)
SD	2 (17)
PD	0 (0)
NE	3 (25)
ORR	7 (58)
DCR	9 (75)

Abbreviations: CR: complete response; PR: partial response; SD: stable disease; PD: progressive disease; NE: not evaluable; ORR: overall response rate; DCR: disease control rate.

## Data Availability

The datasets used and/or analyzed during the current study are available from the corresponding author upon reasonable request.

## References

[1] Curran W.J., Paulus R., Langer C.J., Komaki R., Lee J.S., Hauser S., Movsas B., Wasserman T., Rosenthal S.A., Gore E. (2011). Sequential vs. concurrent chemoradiation for stage III non-small cell lung cancer: Randomized phase III trial RTOG 9410. J. Natl. Cancer Inst..

[2] Yamamoto N., Nakagawa K., Nishimura Y., Tsujino K., Satouchi M., Kudo S., Hida T., Kawahara M., Takeda K., Katakami N. (2010). Phase III study comparing second- and third-generation regimens with concurrent thoracic radiotherapy in patients with unresectable stage III non-small-cell lung cancer: West Japan Thoracic Oncology Group WJTOG0105. J. Clin. Oncol..

[3] Segawa Y., Kiura K., Takigawa N., Kamei H., Harita S., Hiraki S., Watanabe Y., Sugimoto K., Shibayama T., Yonei T. (2010). Phase III trial comparing docetaxel and cisplatin combination chemotherapy with mitomycin, vindesine, and cisplatin combination chemotherapy with concurrent thoracic radiotherapy in locally advanced non-small-cell lung cancer: OLCSG 0007. J. Clin. Oncol..

[4] Sause W.T., Scott C., Taylor S., Johnson D., Livingston R., Komaki R., Emami B., Curran W.J., Byhardt R.W., Turrisi A.T. (1995). Radiation Therapy Oncology Group (RTOG) 88–08 and Eastern Cooperative Oncology Group (ECOG) 4588: Preliminary results of a phase III trial in regionally advanced, unresectable non-small-cell lung cancer. J. Natl. Cancer Inst..

[5] Furuse K., Fukuoka M., Kawahara M., Nishikawa H., Takada Y., Kudoh S., Katagami N., Ariyoshi Y. (1999). Phase III study of concurrent versus sequential thoracic radiotherapy in combination with mitomycin, vindesine, and cisplatin in unresectable stage III non-small-cell lung cancer. J. Clin. Oncol..

[6] Ngwa W., Irabor O.C., Schoenfeld J.D., Hesser J., Demaria S., Formenti S.C. (2018). Using immunotherapy to boost the abscopal effect. Nat. Rev. Cancer.

[7] Gandhi L., Rodríguez-Abreu D., Gadgeel S., Esteban E., Felip E., De Angelis F., Domine M., Clingan P., Hochmair M.J., Powell S.F. (2018). Pembrolizumab plus chemotherapy in metastatic non-small-cell lung cancer. N. Engl. J. Med..

[8] Paz-Ares L., Luft A., Vicente D., Tafreshi A., Gümüş M., Mazières J., Hermes B., Çay Şenler F., Csőszi T., Fülöp A. (2018). Pembrolizumab plus chemotherapy for squamous non-small-cell lung cancer. N. Engl. J. Med..

[9] West H., McCleod M., Hussein M., Morabito A., Rittmeyer A., Conter H.J., Kopp H.G., Daniel D., McCune S., Mekhail T. (2019). Atezolizumab in combination with carboplatin plus nab-paclitaxel chemotherapy compared with chemotherapy alone as first-line treatment for metastatic non-squamous non-small-cell lung cancer (IMpower130): A multicentre, randomised, open-label, phase 3 trial. Lancet Oncol..

[10] Borghaei H., Paz-Ares L., Horn L., Spigel D.R., Steins M., Ready N.E., Chow L.Q., Vokes E.E., Felip E., Holgado E. (2015). Nivolumab versus docetaxel in advanced nonsquamous non-small-cell lung cancer. N. Engl. J. Med..

[11] Theelen W.S.M.E., Chen D., Verma V., Hobbs B.P., Peulen H.M.U., Aerts J.G.J.V., Bahce I., Niemeijer A.L.N., Chang J.Y., de Groot P.M. (2021). Pembrolizumab with or without radiotherapy for metastatic non-small-cell lung cancer: A pooled analysis of two randomised trials. Lancet Respir. Med..

[12] Rahim M.K., Okholm T.L.H., Jones K.B., McCarthy E.E., Liu C.C., Yee J.L., Tamaki S.J., Marquez D.M., Tenvooren I., Wai K. (2023). Dynamic CD8+ T cell responses to cancer immunotherapy in human regional lymph nodes are disrupted in metastatic lymph nodes. Cell.

[13] Antonia S.J., Villegas A., Daniel D., Vicente D., Murakami S., Hui R., Yokoi T., Chiappori A., Lee K.H., de Wit M. (2017). Durvalumab after chemoradiotherapy in Stage III non-small-cell lung cancer. N. Engl. J. Med..

[14] Spigel D.R., Faivre-Finn C., Gray J.E., Vicente D., Planchard D., Paz-Ares L., Vansteenkiste J.F., Garassino M.C., Hui R., Quantin X. (2022). Five-year survival outcomes from the PACIFIC trial: Durvalumab after chemoradiotherapy in stage III non-small-cell lung cancer. J. Clin. Oncol..

[15] Calvert A.H., Newell D.R., Gumbrell L.A., O’Reilly S., Burnell M., Boxall F.E., Siddik Z.H., Judson I.R., Gore M.E., Wiltshaw E. (1989). Carboplatin dosage: Prospective evaluation of a simple formula based on renal function. J. Clin. Oncol..

[16] National Cancer Institute (2009). Common Terminology Criteria for Adverse Events (CTCAE).

[17] Demaria S., Ng B., Devitt M.L., Babb J.S., Kawashima N., Liebes L., Formenti S.C. (2004). Ionizing radiation inhibition of distant untreated tumors (abscopal effect) is immune mediated. Int. J. Radiat. Oncol. Biol. Phys..

[18] Liang H., Deng L., Chmura S., Burnette B., Liadis N., Darga T., Beckett M.A., Lingen M.W., Witt M., Weichselbaum R.R. (2013). Radiation-induced equilibrium is a balance between tumor cell proliferation and T cell-mediated killing. J. Immunol..

[19] Shaverdian N., Lisberg A.E., Bornazyan K., Veruttipong D., Goldman J.W., Formenti S.C., Garon E.B., Lee P. (2017). LeePrevious radiotherapy and the clinical activity and toxicity of pembrolizumab in the treatment of non-small-cell lung cancer: A secondary analysis of the KEYNOTE-001 phase 1 trial. Lancet Oncol..

[20] Antonia S.J., Villegas A., Daniel D., Vicente D., Murakami S., Hui R., Kurata T., Chiappori A., Lee K.H., de Wit M. (2018). Overall Survival with durvalumab after chemoradiotherapy in stage III NSCLC. N. Engl. J. Med..

[21] Deng L., Liang H., Burnette B., Beckett M., Darga T., Weichselbaum R.R., Fu Y.X. (2014). Irradiation and anti-PD-L1 treatment synergistically promote antitumor immunity in mice. J. Clin. Invest..

[22] Postow M.A., Callahan M.K., Barker C.A., Yamada Y., Yuan J., Kitano S., Mu Z., Rasalan T., Adamow M., Ritter E. (2012). Immunologic correlates of the abscopal effect in a patient with melanoma. N. Engl. J. Med..

[23] Dovedi S.J., Adlard A.L., Lipowska-Bhalla G., McKenna C., Jones S., Cheadle E.J., Stratford I.J., Poon E., Morrow M., Stewart R. (2014). Acquired resistance to fractionated radiotherapy can Be overcome by concurrent PD-L1 blockade. Cancer Res..

[24] Palma D.A., Senan S., Tsujino K., Barriger R.B., Rengan R., Moreno M., Bradley J.D., Kim T.H., Ramella S., Marks L.B. (2013). Predicting radiation pneumonitis after chemoradiation therapy for lung cancer: An international individual patient data meta-analysis. Int. J. Radiat. Oncol. Biol. Phys..

[25] Sekine I., Sumi M., Ito Y., Nokihara H., Yamamoto N., Kunitoh H., Ohe Y., Kodama T., Saijo N., Tamura T. (2006). Retrospective analysis of steroid therapy for radiation-induced lung injury in lung cancer patients. Radiother. Oncol..

[26] Shintani T., Kishi N., Matsuo Y., Ogura M., Mitsuyoshi T., Araki N., Fujii K., Okumura S., Nakamatsu K., Kishi T. (2021). Incidence and risk factors of symptomatic radiation pneumonitis in non-small-cell lung cancer patients treated with concurrent chemoradiotherapy and consolidation durvalumab. Clin. Lung Cancer.

[27] Peters S., Felip E., Dafni U., Belka C., Guckenberger M., Irigoyen A., Nadal E., Becker A., Vees H., Pless M. (2019). Safety evaluation of nivolumab added concurrently to radiotherapy in a standard first-line chemoradiotherapy regimen in stage III non-small cell lung cancer—The ETOP Nicolas trial. Lung Cancer.

[28] Peters S., Felip E., Dafni U., Tufman A., Guckenberger M., Álvarez R., Nadal E., Becker A., Vees H., Pless M. (2021). Progression-free and overall survival for concurrent nivolumab with standard concurrent chemoradiotherapy in locally advanced stage IIIA-B NSCLC: Results from the European thoracic oncology platform Nicolas Phase II trial (European thoracic oncology Platform 6–14). J. Thorac. Oncol..

[29] Jabbour S.K., Berman A.T., Decker R.H., Lin Y., Feigenberg S.J., Gettinger S.N., Aggarwal C., Langer C.J., Simone C.B., Bradley J.D. (2020). Phase 1 Trial of Pembrolizumab Administered Concurrently with Chemoradiotherapy for Locally Advanced Non-Small Cell Lung Cancer: A Nonrandomized Controlled Trial. JAMA Oncol..

[30] Jabbour S.K., Lee K.H., Frost N., Breder V., Kowalski D.M., Pollock T., Levchenko E., Reguart N., Martinez-Marti A., Houghton B. (2021). Pembroli-499 zumab Plus Concurrent Chemoradiation Therapy in Patients with Unresectable, Locally Advanced, Stage III Non-Small Cell 500 Lung Cancer: The Phase 2 KEYNOTE-799 Nonrandomized Trial. JAMA Oncol..

[31] Bradley J.D., Hu C., Komaki R.R., Masters G.A., Blumenschein G.R., Schild S.E., Bogart J.A., Forster K.M., Magliocco A.M., Kavadi V.S. (2020). Long-term results of NRG oncology RTOG 0617: Standard- versus high-dose chemoradiotherapy with or without cetuximab for unresectable stage III non-small-cell lung cancer. J. Clin. Oncol..

[32] Senan S., Brade A., Wang L.H., Vansteenkiste J., Dakhil S., Biesma B., Martinez Aguillo M., Aerts J., Govindan R., Rubio-Viqueira B. (2016). PROCLAIM: Randomized phase III trial of pemetrexed-cisplatin or etoposide-cisplatin plus thoracic radiation therapy followed by consolidation chemotherapy in locally advanced nonsquamous non-small-cell lung cancer. J. Clin. Oncol..

